# Impact of Integrase Inhibition Compared With Nonnucleoside Inhibition on HIV Reservoirs in Lymphoid Tissues

**DOI:** 10.1097/QAI.0000000000002026

**Published:** 2019-03-15

**Authors:** Meghan Rothenberger, Krystelle Nganou-Makamdop, Cissy Kityo, Francis Ssali, Jeffrey G. Chipman, Gregory J. Beilman, Torfi Hoskuldsson, Jodi Anderson, Jake Jasurda, Thomas E. Schmidt, Samuel P. Calisto, Hope Pearson, Thomas Reimann, Caitlin David, Katherine Perkey, Peter Southern, Steve Wietgrefe, Erika Helgeson, Cavan Reilly, Ashley T. Haase, Daniel C Douek, Courtney V. Fletcher, Timothy W. Schacker

**Affiliations:** a Department of Medicine, University of Minnesota, Minneapolis, MN;; b Vaccine Research Center, National Institutes of Allergy and Infectious Diseases, NIH, Bethesda, MD;; c Joint Clinical Research Center, Kampala, Uganda;; Departments of d Surgery;; e Microbiology and Immunology; and; f Biostatistics, University of Minnesota, Minneapolis, MN; and; g College of Pharmacy, University of Nebraska Medical Center, Omaha, NE

**Keywords:** HIV, pharmacology, drug levels, antiviral effect, virus decay

## Abstract

**Background:**

HIV is produced in lymphoid tissues (LT) and stored on the follicular dendritic cell network in LT. When antiretroviral therapy is started, plasma viremia decays in 2 phases; the first within days of starting therapy and the second over weeks. Raltegravir (RAL), an integrase inhibitor, has been associated with only a single rapid phase of decay, and we speculated this may be due to higher intracellular concentration (IC) of RAL in LT. We have previously measured suboptimal ICs of antiretroviral therapy agents in LT, which were associated with slower decay of both vRNA+ cells and the follicular dendritic cell network pool.

**Setting:**

Outpatient clinic at the Joint Clinical Research Center in Kampala, Uganda.

**Methods:**

We compared the rate of decay in LT in people starting RAL with those starting efavirenz (EFV).

**Results:**

There was no difference in the rate of virus decay in LT. The ratio of the ICs of RAL and EFV in lymph node to the concentration of drug that inhibits 95% of virus in blood was 1 log lower in lymph node for EFV and >3 logs lower for RAL.

**Conclusion:**

These data further highlight the challenges of drug delivery to LT in HIV infection and demonstrate that RAL is not superior to EFV as judged by direct measurements of the source of virus in LT.

## INTRODUCTION

The decay of plasma HIV RNA typically follows a biphasic pattern in which the half-life of the first phase is about 1 day and that of the second phase about 14 days,^1–3^ with comparable estimates for first-phase decay in SIV-infected rhesus macaques.^4,5^ In lymphatic tissues (LT) where the frequency of HIV RNA (vRNA+) in cells and the number of virions on the follicular dendritic cell network (FDCn) in LT represent the largest virus reservoir in the body,^6^ the rate of decay also occurs in 2 phases.^7,8^ Although substantial evidence supports the current view that first-phase decay reflects the death of activated CD4 T cells infected before antiretroviral therapy (ART) was begun,^8^ the sources of viral RNA in the second phase of decay that have been invoked in mathematical models, or for which there is experimental evidence, include longer-lived infected cells such as macrophages,^2^ resting CD4 T cells,^9^ dissociation of virus from the FDCn,^10^ and productively infected CD4^+^ T cells that are not subject to clearance by host immune responses because of waning levels of HIV antigen.^11^

Raltegravir (RAL) belongs to the class of integrase inhibitors that potently suppress HIV and SIV replication,^12–15^ and has been reported to alter second-phase decay kinetics in a way that challenges the current view that longer-lived infected cells are the source of virus in this phase.^16^ The mathematical modeling of decay of HIV RNA in blood in the data presented by Murray et al^16^ posited the origins of virus in the second phase as arising from cells newly infected by long-lived infected cells, or activation of latently infected cells with full-length unintegrated HIV DNA.

We hypothesized that the data are also quite consistent with the greater efficacy of integrase inhibitors in a particular cell type and/or anatomical compartment such as the LT. Integrase inhibitors might provide better drug levels in LT than other antiretroviral drugs. We have previously shown that tenofovir disoproxil fumarate (TDF), emtricitabine (FTC), atazanavir (ATV), darunavir (DRV), and efavirenz (EFV) do not achieve concentrations in LT's that would be predicted to be fully suppressive.^17^ We tested the major predictions that RAL achieves higher cell-associated concentrations in LT than other ART agents, to thereby accelerate decay of vRNA+ cells and the FDCn pool in LT, in a randomized trial in 11 research participants at the Joint Clinical Research Center in Kampala, Uganda. We compared RAL/TDF/FTC with EFV/TDF/FTC in lymph nodes (LNs) sampled at multiple time points before and during the first 4 months of ART to compare the kinetics of virus decay in LN between treatment groups and to measure cell-associated concentrations of drug in each compartment.

## METHODS

### Plasma Viral Load

Plasma HIV viral load was measured using the COBAS Ampliprep/COBAS TaqMan 96 (Roche, Branchburg, NJ), with a linearity range of 20–10,000,000 copies/mL, or Abbott m2000 system platform, with a linearity range of 40–10,000,000 copies/mL. The Abbott platform was used in the first 12 months of the study and the Roche for all subsequent measures. Both platforms were registered on an external quality assurance program provided by the American Pathologists and Virology Quality Assurance from RUSH University Medical Center.

### CD4 T-Cell Analyses

Blood CD4 cell counts were measured at the same time points by flow cytometry using FACSCount (Becton Dickinson, Inc., Franklin Lakes, NJ).

### In Situ Hybridization

vRNA+ cells were enumerated by using the previously described RNAscope methods.^7,18^ The size of the pool of virions attached to the FDCn was determined by combining in situ hybridization (ISH), as described elsewhere.^6^ In brief, 5–10 sections that were 4-µm thick were cut from different regions of the node, adhered to siliconized glass slides, and deparaffinized. After treatments to facilitate diffusion of the probe, the sections were hybridized to a collection of antisense ^35^S-labeled riboprobes complementary to ∼90% of full-length HIV genomic RNA sequences. After hybridizing and washing, the sections were coated with nuclear track emulsion, exposed for 24 hours, developed, and stained. The probe bound to viral RNA in virions associated with FDCs and their processes generates a diffuse hybridization signal of silver grains scattered over germinal centers. The number of silver grains is proportional to the number of viral RNA copies, and, consequently, the latter can be estimated from the number of silver grains, the specific activity of the probe, the exposure time, and the efficiency of 0.5 grains/dpm for ^35^S. This method of estimating copy number has been validated and is reproducible within ±15%.^6^ Images of autoradiographs, illuminated with epipolarized light, were captured, and silver grains were counted. The number of HIV RNA copies was expressed as log_10_/g LT by estimating the weight of the individual sections from the product of the area (A), thickness (T), and density (D) of the section (A × T × D).

### Quantitative Image Analysis

Photographic images were captured, and the frequency of vRNA+ or vDNA+ cells was measured and expressed as the total per unit area. These methods have been extensively reviewed.^6,19,20^

### Measures of Drug Concentration

Cell-associated concentrations of tenofovir-diphosphate (TFV-DP), FTC-triphosphate (FTC-TP), lamivudine-TP (3TC-TP), EFV, and RAL were quantified from lysed cellular matrix from peripheral blood mononuclear cells (PBMCs), and MNCs obtained from biopsy samples of the LN and rectum (RALT) using methods previously described.^17,21^ Final sample extracts were quantified with a liquid chromatography–triple quadrupole mass spectrometer system consisting of a Shimadzu Nexera ultra high-performance liquid chromatograph attached to an AB Sciex 5500 QTrap mass spectrometer. Quality control sample interbatch coefficients of variance for the TFV-DP, FTC-TP, and 3TC-TP, and the EFV and RAL methods were 3.84%–7.67% and 1.1%–7.4%, respectively. Absolute mean relative errors to the theoretical target quality control samples were <5.6% for TFV-DP, FTC-TP, and 3TC-TP, and <8.1% for EFV and RAL. Batch acceptance criterion was derived according to the Food and Drug Administration Guidance for Industry on Bioanalytical Method Validation.^22^ Analytical results were expressed in femtomoles per million cells. Relative penetration of the ARVs into the LN and RALT was assessed as a ratio of concentrations to those in PBMCs.

### Statistical Analysis

Linear random effects models with subject-specific intercepts were used for each analysis. For the comparison of trends over time between the 2 treatment groups, only data up to 3 months were used and the analyses were conducted using models with fixed effects for time, RAL treatment group, and the interaction between RAL treatment group and time. To compare differences in drug concentration among the LN, PBMC, and rectum, drug concentrations recorded below the level of detection were imputed using the smallest observable value for that drug. Drug concentrations were log base 10 transformed before model fitting. Only time points that had recorded drug concentrations for all 3 locations were used in the analysis. The analyses were conducted using separate models for each drug with fixed effects for each location (LN, PBMC, and rectum). Because of the small sample size, permutation *P* values are presented for all models.

### Informed Consent

Patients were recruited at the Joint Clinical Research Center using protocols and consent forms that were approved by the University of Minnesota IRB and also approved by the IRB at the Joint Clinical Research Center in Kampala, Uganda, and the Uganda National Council of Science and Technology (UNCST). All subjects were adults and provided informed written consent. The clinical trial occurred before the requirement for CT.gov registration.

## RESULTS

### Cohort Description and Clinical Trial

We recruited a cohort of 11 research participants at the Joint Clinical Research Center (JCRC) in Kampala, Uganda. Participants were required to be HIV+ with detectable plasma HIV viremia and have no history of prior ART use. We enrolled a total of 4 men and 7 women with chronic HIV infection whose mean age was 34.6 years (range 24–44 years) with an average peripheral CD4 T-cell count of 396 cells/µe (range 204–985 cells/µL). The mean plasma viral load (pVL) at entry was 215,954 copies/mL (range 5420–755,930 copies/mL).

Individuals were randomized to receive either EFV (600 mg/d) or RAL (400 mg twice daily) along with FTC (200 mg/d) plus TDF (300 mg/d) combined into one tablet [ie, Truvada (TRV)]. After 3 months of the assigned ART, all subjects were switched to an open label regimen designated by the national protocol standard for Uganda at the time of the study (usually EFV and either FTC plus TDF or 3TC plus TDF), and they were followed up for an additional 3 months. At baseline, before initiation of ART, an inguinal LN was obtained^23^ and again 2 and 7 days later, and then 4 months after the start of ART. A rectal biopsy was obtained at the 4-month time point. Peripheral blood was sampled for measures of pVL at baseline and again at day 2, day 7, day 14, month 3, month 4, and month 6 after initiating ART.

### HIV RNA+ RNA Decay in PBMC, LNMC, and B-Cell Follicles

We measured pVL and the amount of virus in the inguinal LN with ISH using a mix of primers validated for detection of HIV clades A and D, the predominant clades found in Uganda. We analyzed 4-µm sections of formalin-fixed, paraffin-embedded tissues, evaluating every fifth section in at least 5 sections in total to provide analysis through ≥ 80 µm of each tissue. The frequency of HIV RNA+ cells was determined in each section and converted to the frequency per gram (g) from the measured area of the section, nominal thickness, and previously determined density of fixed tissue of ∼ 1 g/cm^3^.^24^ For example, the frequency of vRNA+ cells/µm^2^ area × 4-µm thick = vRNA+ cells/µm^3^ × 10^12^ µm^3^/cm^3^ × 1gm/cm^3^ = vRNA+ cells per gram of tissue.

**FIGURE 1.**
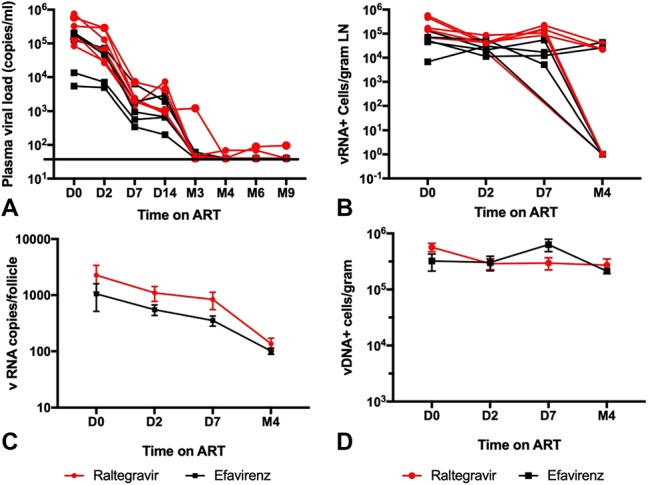
Change in pVL (A), vRNA+ cells/g in the LN (B), the decay of virus off of the FDCn (C), and changes in the frequency of vDNA+ cells in the LN (D) between the group receiving RAL/FTC/TDV (gray) and the group receiving EFV/FTC/TDV (black).

Using these methods, we found no difference between treatment groups in the rate of decay of HIV RNA in plasma (Fig. 1A and Table 1, *P* = 0.356), vRNA+ cells/g LN (Fig. 1B and Table 1, *P* = 0.365), the rate of decay of virus off of the FDCn of B-cell follicles (Fig. 1C and Table 1, *P* = 0.856), or in decay of vDNA+ cells/g LN (Fig. 1D and Table 1, *P* = 0.189). Thus, by any viral measure we performed, we did not detect a difference in the rate of decay between RAL- and EFV-containing regimens. Of note, we did detect vRNA+ cells in 5/11 (45%) of participants in the LN of the month 4 time point consistent with other cohorts we have studied with a 6-month follow-up on ART.^17^ There was no association between treatment regimen or detection of vRNA+ cells (3 in the RAL arm and 2 in the EFV arm).

**TABLE 1.**
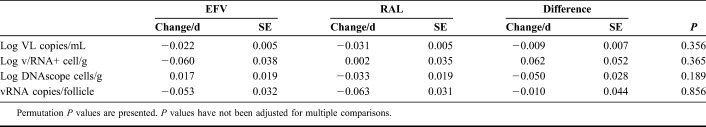
Difference in Change in VL Copies, Ki67 LN Cells, vRNA+ Cell, DNAscope Cells, and vRNA Copies/Follicle (Until M3) Between the Two Treatment Groups (RAL, TDF, FTC vs. EFV, TDF, 3TC)

### Decreased Concentration of ART in the LN and Rectum Compared With PBMC

We measured the cell-associated concentrations of antiretroviral drugs (ARVs) at multiple time points; in PBMC and LN, we measured cell-associated concentrations of drugs each time an LN was removed (ie, days 2 and 7 and month 4), and in the rectum, we measured at the month 4 biopsy time point. We obtained an additional sample of PBMC at the 6-month time point. The median (and interquartile range) cell-associated concentrations in PBMC, LNMC, and rectal mononuclear cells are listed in Table 2. Examining these drug concentrations among the 3 anatomical compartments (LN, PBMC, and rectum) shows that concentrations were most variable for EFV, TFV-DP, and 3TC-TP (Table 2). The cell-associated concentrations of EFV in PBMC were significantly higher than those in the LN and the rectum. RAL cell-associated concentrations were significantly higher in the rectum than the LN, but no significant difference was found between the concentrations in the rectum and PBMC. TFV-DP had significantly different concentrations across the 3 locations with highest levels found in the rectum and lowest levels found in the LN. 3TC-TP concentrations were found to be significantly higher in the PBMC than in the LN or the rectum. The concentrations of FTC-TP were numerically higher in the PBMC than in the LN and rectum.

**TABLE 2.**
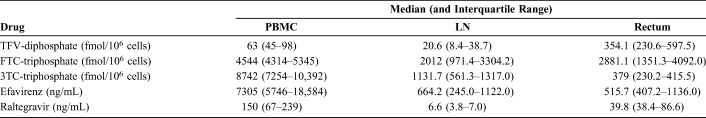
Cell-Associated Concentrations in PBMCs and in the LN and Rectum

In all cases, the median cell-associated concentrations of drug in LT were less than the simultaneous measure in the PBMC, except TFV-DP in the rectum (Table 2). Comparisons of the intraindividual differences in mean concentrations between PBMC and LN demonstrated a significant reduction for EFV (*P* < 0.001), TFV-DP (*P* = 0.006), and 3TC (*P* = 0.007), but not for RAL (*P* = 0.083) or FTC-TP (*P* = 0.103). A similar comparison for the PBMC and rectum demonstrated a significant difference in concentrations with a decrease for EFV (*P* < 0.001), an increase for TFV-DP (*P* = 0.003), and a decrease for 3TC-TP (*P* < 0.001), but no significant change for RAL (*P* = 0.421) or FTC-TP (*P* = 0.080). Comparing the LN with rectum demonstrated a significant difference in RAL (higher in the rectum, *P* = 0.003) and TFV-DP (higher in the rectum, *P* < 0.001); otherwise, there were no significant differences.

**FIGURE 2.**
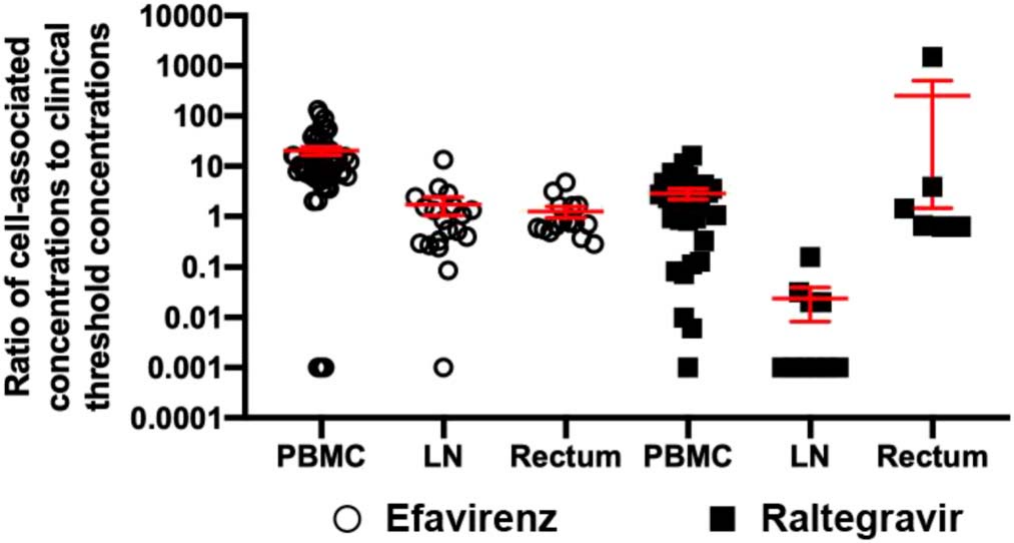
The ratio of the cell-associated concentration of drug measured in each compartment to the putative clinically relevant threshold concentration.

Relationships have been observed for both EFV and RAL between plasma concentrations and virologic response in patients. Such in vivo effective concentrations also provide a basis to compare potential antiviral activity across different ARVs. For EFV, the usually quoted concentration threshold is 1000 ng/mL, although recent results from the ENCORE study provide support that the level could be lower, and at least one study has found 700 ng/mL to be the limit for prediction of virologic suppression.^25,26^ Insight into concentration and effect relationships for RAL can be found in a study of once- vs. twice-daily dosing. Superior rates of virologic suppression were found with twice-daily dosing; although a concentration–effect relationship was clearly seen with once-daily dosing, it was not observed with twice, suggesting that those recipients had concentrations on the plateau portion of the concentration–effect relationship. The geometric mean RAL trough concentration in the lowest quartile of twice-daily dosing was 60 ng/mL.^27^ Based on these clinical data for EFV and RAL, we chose to compare cell-associated concentrations in PBMCs, LN, and rectum with putative clinical threshold concentrations of 700 ng/mL for EFV and 60 ng/mL for RAL. In Figure 2, we compare the ratio of cell-associated concentrations of EFV and RAL in all 3 compartments (PBMC, LN, and rectum) to these clinical threshold concentrations. We find that EFV achieves a median 10.1-fold increase over the threshold in PBMC, but in LN, the median ratio is 0.95. For RAL, the median ratio in PBMCs is 1.5 and in LN is 0.03.

## DISCUSSION

We have previously demonstrated that the decay of virus off of the FDCn is biphasic with a rapid first phase and a longer second phase^8^ that mirrors decay of plasma viremia in PB^1,2,28^ and reflects the loss of first, the activated CD4^+^ T cells and second, infected resting CD4^+^ T cells^8^ that are the sources of virus deposited on the FDCn with half-lives, respectively, of 1 and 14 days.^7^ Given the early reports of an altered pattern of viral decay in plasma occurring with administration of raltegravir,^16^ we thought it possible that raltegravir may alter the decay of vRNA+ cells or the FDCn pool of virus because of higher concentrations in LT. We tested this hypothesis in a randomized trial in ART-naive people in Uganda who received TDF and FTC, plus either RAL or EFV. We sampled LT and blood at frequent intervals to measure changes in pVL, frequency of vRNA+ and vDNA+ cells, and rate of decay off the FDCn over a 4-month follow-up period, and found no difference in these measures. We thus conclude that RAL is essentially equivalent to EFV in virus decay with ART, even in PB. Therefore, we did not replicate findings from Murray et al.^16^ Reasons for this difference are not clear, but other groups have also shown that RAL therapy does not eliminate biphasic decay.^29^

We also measured plasma and cell-associated concentrations of ARVs in the PBMC, LN, and rectum, and again^17^ found significant differences among the PBMC-associated concentrations of drug and the amount simultaneously measured in LN and rectal mononuclear cells. Furthermore, when we referenced those measured concentrations to putative clinical threshold concentrations, we found that this ratio, as a metric of antiviral activity, was less than 1 in the LN and rectum for both EFV and RAL. This observation may provide a pharmacologic basis to explain why there was no difference in the rates of viral decay in LT with EFV- and RAL-based ART.

The consistent observation that there are suboptimal concentrations of ARVs in LT, where 99.6% of the reservoir resides, offers a potential mechanism for the finding of ongoing virus production in a significant proportion of people on long-term ART.^6,17,30,31^ In well-suppressed SIV+ macaques on long-term ART or HIV+ humans, we have shown that virus producing cells persist, using a technique where we combine ISH with tyramine signal amplification using a fluorescent marker (ELF97) to identify virions around the vRNA+ cell,^32^ and there was good agreement between the frequency of vRNA+ cells and the frequency of these cells containing viral particles (Figure 5 and supplementary Table 4 in Ref. 6), again consistent with continuing virus production on ART. The clinical importance and relevance of the persistent detection of these cells, regardless of their ability to make viral particles, while on “suppressive” ART is unknown. It is well recognized that tissue and serum markers of immune activation (IA) decrease during ART for HIV infection; they do not normalize and this persistent IA is linked to increased morbidity and mortality.^33–37^ Concomitant herpes virus or other infections,^38,39^ microbial translocation,^40,41^ and ART-induced metabolic abnormalities^42^ have all been hypothesized to cause or contribute to sustained IA during ART. However, HIV itself has also been proposed to be a driver of IA. Our data are consistent with a hypothesis that despite apparent complete virus suppression measured in peripheral blood, suboptimal LT levels of ARVs enable low levels of virus production that triggers and sustains IA. Further work should be performed to explore this hypothesis.

In summary, our hypothesis that RAL concentrated better in LT to accelerate suppression of virus production and decay of virus-infected cells and virus associated with the FDCn pool was not supported, as we could find no difference in decay between RAL and EFV, in any compartment. Indeed, we show, to the contrary, that there is low penetration of RAL into lymphoid compartments. Furthermore, the finding of persistent detection of vRNA+ cells in this context is consistent with a model of persistent virus production in the setting of suboptimal drug levels. To eliminate virus reservoirs to achieve a HIV cure, these data argue the need for ARVs or formulations of ARVs that penetrate into the LT at concentrations that will completely suppress virus replication.
